# Whole genome sequencing revealed genetic diversity, population structure, and selective signature of Panou Tibetan sheep

**DOI:** 10.1186/s12864-023-09146-2

**Published:** 2023-01-28

**Authors:** Huibin Shi, Taotao Li, Manchun Su, Huihui Wang, Qiao Li, Xia Lang, Youji Ma

**Affiliations:** 1 College of Animal Science and Technology, Gansu Agricultural University, Lanzhou, 730070 China; 2 Gansu Key Laboratory of Animal Generational Physiology and Reproductive Regulation, Lanzhou, 730070 China; 3 Institute of Animal & Pasture Science and Green Agriculture, Gansu Academy of Agricultural Science, Lanzhou, 730070 China

**Keywords:** Panou Tibetan sheep, Population structure, Positive selection, Fst and π ratio

## Abstract

**Background:**

The detection of selective traits in different populations can not only reveal current mechanisms of artificial selection for breeding, but also provide new insights into phenotypic variation in new varieties and the search for genes associated with important traits. Panou sheep is a cultivated breed of Tibetan sheep in China with stable genetic performance, consistent appearance and fast growth and development after decades of artificial selection and cultivation. Due to long-term adaptation to the high altitude, cold and hypoxic environment in the plateau area, they may have formed a unique gene pool that is different from other Tibetan sheep breeds. To explore the genetic resources of Panou sheep, we used next-generation sequencing technology for the first time to investigate the genome-wide population structure, genetic diversity, and candidate signatures of positive selection in Panou sheep.

**Results:**

Comparative genomic analysis with the closely related species Oula sheep (a native breed of Tibetan sheep in China) was used to screen the population selection signal of Panou sheep. Principal component analysis and neighbor joining tree showed that Panou sheep and Oula sheep had differences in population differentiation. Furthermore, analyses of population structure, they came from the same ancestor, and when K = 2, the two populations could be distinguished. Panou sheep exhibit genetic diversity comparable to Oula sheep, as shown by observed heterozygosity, expected heterozygosity and runs of homozygosity. Genome-wide scanning using the Fst and π ratio methods revealed a list of potentially selected related genes in Panou sheep compared to Oula sheep, including histone deacetylase 9 (*HDAC9*), protein tyrosine kinase 2 (*PTK2*), microphthalmia-related transcription factor (*MITF*), vesicular amine transporter 1 (*VAT1*), trichohyalin-like 1 (*TCHHL1*), amine oxidase, copper containing 3 (*AOC3*), interferon-inducible protein 35 (*IFI35*).

**Conclusions:**

The results suggest that traits related to growth and development and plateau adaptation may be selection targets for the domestication and breeding improvement of Tibetan sheep. This study provides the fundamental footprints for Panou sheep breeding and management.

**Supplementary Information:**

The online version contains supplementary material available at 10.1186/s12864-023-09146-2.

## Background

In China, according to the geographical distribution of sheep and their genetic origin, the native sheep are divided into three ancestral populations: Mongolian sheep, Kazakh sheep and Tibetan sheep [1]. Tibetan sheep are the most common, abundant and widely distributed livestock on the Tibetan plateau, and they have spread with human migrations [2]. Domestication during the Neolithic agricultural revolution made a major contribution to human civilization by providing a stable source of meat, wool, leather, and dairy products. Early archaeological and genetic studies provide strong evidence that domestic sheep were domesticated from their wild ancestors in the Fertile Crescent about 12,000–10,000 years BP in the Asian Mouflon [3]. The development of genome sequencing technology is an effective method to reveal the domestication history [4], environmental adaptations [5] and selection characteristics [6] of livestock. Hu et al. [4] found that the distribution of Tibetan sheep in the northeastern and central regions of the Tibetan Plateau was gradually migrating, starting to spread to the southwestern region about 3100 years ago and to the central region 1300 years ago, through whole genome resequencing analysis of Tibetan sheep. The date and route combined with archaeological evidence reveal the history of human expansion along the Tangbo Ancient Road during the late Holocene [4].

The extensive variation in local and improved breeds was the basis for genomic variation, adaptive traits and important agronomic traits in domestic sheep [7, 8]. Thus, sheep have gained worldwide distribution and developed into many unique breeds through adaptation to different environments and genetic improvement under different production systems [8]. Due to the continuing influence of natural selection and artificial breeding, the breeding of sheep breeds requires selection according to the natural environment of sheep and human needs, resulting in breeds of different types than wild populations. The Oula sheep is a local breed of Tibetan sheep. By comparing the Oula sheep with other Tibetan sheep breeds, it has been found that the Oula sheep has obvious differentiation characteristics from other Tibetan sheep [9]. The breeding of the Panou sheep is a new breed group carefully cultivated by breeding experts after 14 years, with Oula sheep as the female parent and wild Argali sheep in the Qinghai area as the male parent. The genomic aggregation breeding technology using this breed selection and molecular marker-assisted selection and appropriate introduction of genes from the wild relatives of Oula sheep (Argali sheep) was used to construct an open joint breeding system of core group with bi-directional gene flow. The specific breeding process is as follows: firstly, two generations of the female parent Oula sheep breed were selected. And then, with the introduction of the argali sheep gene in the 3rd generation, the introduction of the Argali sheep gene in the 4th generation stepwise, and the expansion of the breeding population by the 5th generation cross-fixed. After six generations of pure breeding, a new breed group of Panou sheep with stable genetic performance and consistent external characteristics was initially bred by the end of 2020. Both the Panou (PO) sheep population and the Oula (OL) sheep population belong to the pedigree of Tibetan sheep, but the breeding history of the two breeds is different. According to research, the Oula sheep was formed by the continuous hybridization of wild Argali and Tibetan sheep in the early days [10]. The name of the Oula sheep comes from the “Oula Mountain” that borders Maqu County, Gannan Tibetan Autonomous Prefecture, Gansu Province, Huangnan Tibetan Autonomous Prefecture, Qinghai Province, and Henan Mongolian Autonomous County [11]. The breeding of Panou sheep was based on Oula sheep as the female parent and wild Argali sheep as the male parent, using molecular marker-assisted selection and genomic aggregation breeding techniques. After decades of selection and breeding, the number of Panou sheep has reached 1 million and its genetic performance is gradually stabilized, but the research on the genomic information of Panou sheep is still a gap in China.

By comparing with the genome of its close relative Oula sheep, the genome characteristics of Panou sheep can be determined and whether it has become an independent Tibetan sheep breed. The population differentiation index (Fst) is a measure of the degree of differentiation between two populations [12]. Fst detection for inter-population selection signal analysis was originally used in human evolutionary genetics [13], and now it has been widely used in the study of the molecular genetic mechanism of livestock domestication and the formation of important economic traits [14].

Although SNP arrays have been widely used to identify genetic variants, their results are limited by the low density of probes [15]. High-throughput sequencing allows for improved characterization of genomic features and population structure at the genome-wide level [16]. Next-generation genome sequencing technology has been widely used to study different traits of selection in a variety of species, and to identify genes that affect production traits and livestock domestication-related genes. Candidate genes contributing to environmental adaptation have been identified in humans [17], cattle [18], pigs [19], chickens [20], sheep [21] and goats [22], and some studies on selective signatures genes associated with domestication and production traits have been identified. Chen et al. found that *MITF* was an important gene in the adaptive progressive penetration of Argali sheep to Tibetan sheep [14]. Furthermore, Hu et al. found that MITF could be a candidate gene for studying sheep adaptation to high altitude [4], and this gene was associated with UV resistance [23].

However, to our knowledge, the genome-wide genetic characterization of Panou sheep has not been investigated. In this study, we obtained the whole genome sequencing data of Panou sheep for the first time to deeply understand its genetic diversity and population structure. Comparative genomic analysis with the closely related species Oula sheep was used to screen the population selection signal of Panou sheep, and it could provide theoretical basis for future improvement of economic traits and development of new breeding strategies in Panou sheep.

## Materials and methods

### Samples collection and DNA extraction

Blood samples were collected from the Magu grassland in the area of Oula Town (Longitude: 101.73, Latitude 34.08), Magu County, Gannan Tibetan Autonomous Prefecture, Gansu Province, China **(**see Additional file 1: Table S1). The sheep populations used for the study all exceeded 300 individuals and were distributed in two locations in the Magu grassland. We randomly selected 10 sheep from each of population for the study. With anesthesia, approximately 10 ml of blood was collected from the jugular vein of each animal, placed in vacuum container tubes containing EDTA as an anticoagulant, and stored in liquid nitrogen (− 196 °C). Genomic DNA from the whole blood was extracted using Genomic DNA Purification Kit (Thermo Fisher Scientific Inc., Waltham, MA, USA). The concentration and purity of DNA were measured by Nano-Drop 2000 spectrophotometer (Thermo Fisher Scientific Inc., Waltham, MA, USA) and stored at − 20 °C for Whole-Genome Sequencing [5].

### Library construction and whole genome sequencing

The genomic DNA samples were subjected to biological digestion with the restriction endonuclease, followed the Paired-End DNA Sample Prep kit (Illumina Inc., San Diego, CA, USA), with a process of end repaired, dA-tailed, ligated to paired-end adaptors and PCR amplification with 300-bp inserts. The constructed libraries were sequenced using Illumina HiSeq X Ten (Illumina Inc., San Diego, CA, USA) NGS platform at Genedenovo company (Guangzhou, China) to obtain 150 bp (PE150) paired-end read data. All samples were sequenced at 10× coverage. Quality trimming was an essential step to generate high confidence of variant calling. Raw reads were processed to get high quality clean reads according to three stringent filtering standards: 1) removing reads with ≥ 10% unidentified nucleotides (N); 2) removing reads with > 50% bases having phred quality scores of ≤ 20; 3) removing reads aligned to the barcode adapter [24].

### Sequence alignment and variant calling

The program Burrows-Wheeler Aligner [25] (BWA, version 0.7.12, BWA-MEM algorithm) was then used to map the clean reads to the Tibetan sheep reference genome CAU_O.aries_1.0 (https://www.ncbi.nlm.nih.gov/assembly/GCA_017524585.1) with the default parameters (−mem 4 -k 32 -M). The mapped reads were further filtered by removing multiply aligned reads, and the remaining high-quality reads were kept for variant calling. We used the software Genome Analysis Toolkit (GATK, version 3.4–46) [26] with the parameters (−Window 4，-filter “QD < 2.0 || FS > 60.0 || MQ < 40.0 “，-G_filter “GQ < 20”) to call SNPs according to previous recommendations.

The markers obtained from the process analysis need to be further filtered. VCFtools-0.1.14 [27] with the parameters (−vcf filter_idv.recode.vcf -maf 0.05 \ -recode -recode-INFO-all -out maf05) was used to remove the indel in the markers, and then PLINK 1.9 [28] with the parameters (−file a -geno 0.1 -recode -out re) was used to filter the SNP sites. We used PLINK 1.9 software for quality control (QC) of these SNPs. SNPs with call rate 95%, MAF < 0.05 and heterozygosity > 0.2 were filtered out. In addition, more than 10% of the samples missing genotyping were deleted from the dataset. Moreover, to minimize the influence of sequencing and mapping bias, variant sites with QD < 2.0; FS > 60.0; MQ < 40.0; Haplotype Score > 13.0; Mapping Quality Rank Sum < − 12.5; Read Pos Rank Sum < − 8.0 were discarded. Sites showing an extremely low (< 2×) or high average coverage (> 15×) were also filtered out. Following the SNP calling, the software tool ANNOVAR 2.0 [29] with the parameters (−geneanno -dbtype refGene) was used for SNP annotations.

### The analysis of homozygosity and population history

We used the -homozyg function in the PLINK 1.9 program [28] with the parameters (−homozyg homozyg-density 10 homozyg-gap 100 homozyg-kb 100 homozyg-snp 10 homozyg-window-het 1 homozyg-window-missing 5 homozyg-window-snp 50 homozyg-window-threshold 0.05) to identify and characterize the ROH of two sheep breeds. The degree of ROH variation between populations was characterized based on differences in the length and number of ROH fragments between populations. The genomic inbreeding coefficient F_ROH_ is an important parameter to evaluate the degree of inbreeding among populations. The calculation method is as follows: ${F}_{ROH}=\sum \frac{L_{ROH}}{L_{AUTO}}$. Among them, L_ROH_ refers to the total length of ROH of each individual in the genome, and L_AUTO_ is the specific length of the autosomal genome covered by the SNPs of whole-genome sequencing. The incidence of adjacent SNPs and ROHs exceeds a threshold in genomic regions forming so-called Island [30]. On a population basis, the proportion of each SNP site falling within the ROH (ROH ratio) was calculated. Manhattan plots were drawn based on the ROH ratios of SNP loci. The ROH ratio of top 1% [31] was taken as the threshold line of high-frequency SNPs, and the ROH island is obtained according to the distribution of SNP sites exceeding the threshold in the genome.

We identified the most common genomic regions and candidate genes associated with ROH in two Tibetan sheep breeds by assessing the ROH Island shared between individuals, and plotted Manhattan plots against the ROH ratio for each SNP locus.

Using PSMC [30] (with the parameters: -p 4 + 25*2 + 4 + 6 -r 4 -t 15 -N 30) and SMC++ [32] (with the parameters: -p 0.5 -m 2.5e-8 -w 100 -em 20 -sp cubic) two methods to infer the effective population size (Ne) based on a single fully sequenced diploid individual dynamic changes. For PSMC/SMC++ analysis, scaling was performed using a neutral mutation rate u = 2.5 × 10^− 8^ and a generation time of 2 years. To assess genomic linkage disequilibrium (LD) patterns in different populations, we utilized Haploviewb 4.2 [33] program estimated allele frequency correlation (r^2) with the following parameters: -maxdistance 1000 -dprime -minGeno 0.6 -minMAF 0.05 -hwcutoff 0, and plot the LD decay using the R script.

### Population structure analysis

Using the filtered SNP sets, performing population structure analysis: (1) Population structure analysis based on all SNP information using ADMIXTURE V 1.3.0 software [34] (for detailed parameters please see http://dalexander.github.io/admixture/), and the number of ancestral clusters (K) was set from 1 to 9, and five-fold cross-validation was run to determine the cross-validation error Minimum K value. (2) Principal Component Analysis (PCA) was performed using PLINK 1.9 [28], and the first four principal components affecting variation in the dataset were identified, and the first two dimensions were used to distinguish population structure. (3) Using ROH markers as SSR markers, using Ape software [35], the phylogenetic tree was constructed according to the neighbor joining method.

### Selective sweep analysis

Based on filtered SNPs, using PopGenome software [36, 37]，sliding window by physical length, with 100 kb as the window and 10kp as the step, to analyze the nucleotide diversity within the population, the neutrality test, and the diversity comparison between the populations. Fixation index, also known as genetic differentiation index, or Fst analysis [38], the degree of differentiation between populations can be calculated based on genetic information. The formula for calculating Fst is as follows: ${F}_{st}=\frac{\pi_{Between}-{\pi}_{Within}}{\pi_{Between}}$. Among them, π_Within_ represents the average number of individual differentially paired bases within the group, and π_Between_ represents the average number of individual differentially paired bases between groups. The top 1% was selected as the significance threshold of Fst.

Nucleotide diversity represents to some extent the genetic diversity of a population, and π analysis [39] is a method that uses SNPs to calculate the average difference between any two nucleotide sequences in a population. And then analyzes the genetic diversity within the population by comparing the change in π values, where the π value is the average of pairwise differences in nucleic acid sequences; the θ watterson value is then calculated based on the number of segregation sites of all nucleic acids [40]. As the name implies, it refers to nucleotide diversity. The higher the value, the higher the nucleotide diversity. It is commonly used to measure nucleotide diversity within a population and can also be used to infer evolutionary relationships. The π value is calculated based on the sliding window method and displayed using the Manhattan chart. It can be intuitively seen in which genome interval the π value of the population is significantly reduced. The formula for calculating the value of π is as follows:

$$\uppi =\sum_{ij}{x}_i{x}_j{\pi}_{ij}=2\ast \sum_{i=2}^n\sum_{j=1}^{i-1}{x}_i{x}_j{\pi}_{ij}$$

Among them, x_i_ and x_j_ represent the frequency of the i-th and j-th sequences, respectively, π_ij_ represents the number of base differences between the i-th and j-th sequences of individuals in the same population, and n represents the total number of sequences in the sample.

π ratio analysis [41], another way to determine the signal of selection，mainly focuses on the fold difference in diversity of a locus (or interval) between two subpopulations. Compared with Fst, which is concerned with the differentiation of genotypes, π ratio is concerned with the change of diversity value. In π ratio, the denominator population is the selection population, and the numerator population is the control population. After calculating the nucleotide diversity (π) results based on the sliding window strategy, take the ratio according to the control/selection method, and then use the Manhattan plot to display the ratio, which can intuitively see the distribution of the π ratio between the two groups. The calculation formula of π ratio is as follows: π ratio = π(A)/π(B) where π(A) and π(B) are the π values of the control group and the selection group, respectively. The top 1% is selected as the significance threshold of π ratio.

### Functional enrichment analysis of candidate genes

After the candidate genes in the selected regions were obtained by combining the Fst and π ratio values, Gene Ontology (GO) and Kyoto Encyclopedia of Genes and Genomes (KEGG) analysis were performed on these genes to understand the approximate functions of these genes and provide clues for further screening and verification. All candidate genes were mapped to terms in the GO and KEGG databases, and the number of significant genes for each term was determined with a *P* value ≤0.05 as a threshold. Terms satisfying this criterion were defined as GO and KO terms that were significantly enriched for candidate genes.

## Results

### Whole-genome sequencing and genetic variation

We used Illumina Hiseq X10 PE150 sequencing platform to perform whole-genome resequencing of 20 Tibetan sheep individuals, and obtained more than 600 Gb, 2 × 150 bp end sequences. The average sequencing depth of each species was 11.57× (OL) and 11.73× (PO) using Burrows-Wheeler Aligner (BWA) [25] software aligned the resequencing data to Tibetan sheep reference genome, and finally identified 25,821,514 SNPs and 2,268,652 indels. To improve accuracy of the analysis, we filtered original SNPs by the deletion of the loci, and used filtered markers for selection pressure analysis. The filter condition is: removing marker sites with a missing rate exceeding 50%. The number of SNPs used for analysis in these two groups was 22,436,213 (OL) and 21,267,961 (PO), respectively, overall π values were 0.0027 (OL) and 0.0026 (PO), for subsequent analysis. We obtained high-quality SNPs and indels from 20 re-sequenced sheep, most of which were located in the intergenic region (18,119,221, 64.50%), only 0.60% (167,393) were located in the exonic region, and 33.75% in the proportion of the intron region (see Additional file 2: Table S2**)**. A total of 65,218 non-synonymous sites (38.96%) and 96,252 synonymous sites (57.50%) were located within exons, resulting in a non-synonymous/synonymous ratio of 0.68 (see Additional file 3: Table S3**)**. All raw data were deposited in the NCBI BioProject chapter with accession number PRJNA797957.

Effective population size inference for sheep populations is based on analysis of ROH, F_ROH_ and LD decay. According to the set parameters, a total of 740 ROHs were detected in the 2 sheep breeds, with an average of 37 detected per sample. On average, ROH (392) was higher in OL than PO (348) (means 0.35 Mb and 0.36 Mb, respectively). The minor allele frequencies were almost similar between the two breeds (Table 1).

**Table 1:** The level of genetic ROH the two populations

Breed name	Acronym	Sample Size (n)	Number of ROH	Length of the Genome (Mb)	Mean (Mb)	MAF < 0.05
OuLa	OL	10	392	836.61	0.35	0.2235
PanOu	PO	10	348	752.1	0.36	0.2283

**Fig. 1:**
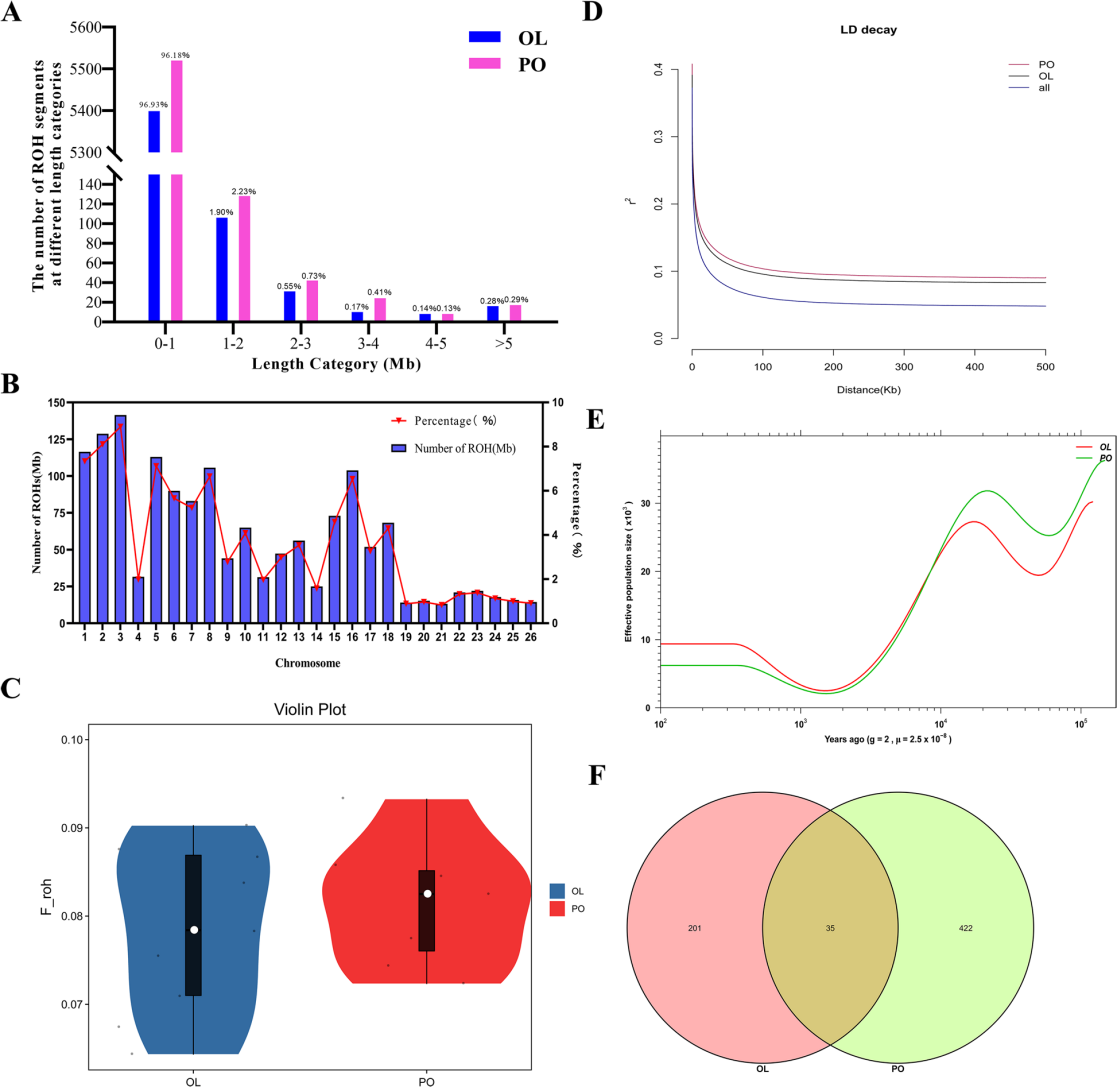
Information about ROH, inbreeding coefficient (F_ROH_), LD decay, and effective population size. **A** The number of ROH segments in different length categories. **B** The distribution of the length and number of ROH in different individual. The X-axis denotes the number of ROHs per chromosome and the Y-axis represents the average percentage (%) of each chromosome covered by ROH (red lines). **C** Violin plot of genomic inbreeding coefficient in two sheep populations. **D** Linkage disequilibrium decay (LD decay) in two breeds. **E** Estimated effective population sizes (Ne) over time for two breeds. **F** Analysis of common genes of two Tibetan sheep breeds ROH island

To compare the distribution of ROH across breeds, we divided the ROH length into 6 categories (0–1, 1–2, 2–3, 3–4, 4–5 and > 5 Mb). More than 96% of ROH lengths in both varieties were in 0-1 Mb range, and the relative number distribution of ROH with different lengths was shown in Fig. 1A. With an average length of 2.15 Mb, the longest fragment detected between varieties on the OAR3 chromosome was 141.44 Mb, containing 94,171 SNPs, followed by the OAR2 chromosome, which contained 128.76 Mb and 100,496 SNPs. Overall, the total number of ROHs per chromosome decreased with decreasing chromosome length. The highest percentage of ROH per chromosome was for chromosome OAR3 (8.90%) and chromosome OAR2 (8.10%), while the lowest length was for chromosome OAR21 (0.82%) (Fig. 1B). The inbreeding coefficient of the OL sheep population was lower than that of the PO sheep breed (F_ROH_PO_ = 0.081, F_ROH_OL_ = 0.078, Fig. 1C).

Despite the relatively low valuation of Ne (PO: 6212; OL: 9367) [4], the SMC++ results for the samples (Fig. 1E) showed consistent population trends inferred from PSMC (see Additional file 4: Fig. S1**)**. Linkage disequilibrium (LD) decay analysis showed that the breeding histories of the two Tibetan sheep breeds were significantly different. Within the genetic distance interval, LD decay was higher in OL sheep and slower in PO sheep **(**Fig. 1D**)**.

### Runs of homozygosity (ROH) patterns

**Fig. 2:**
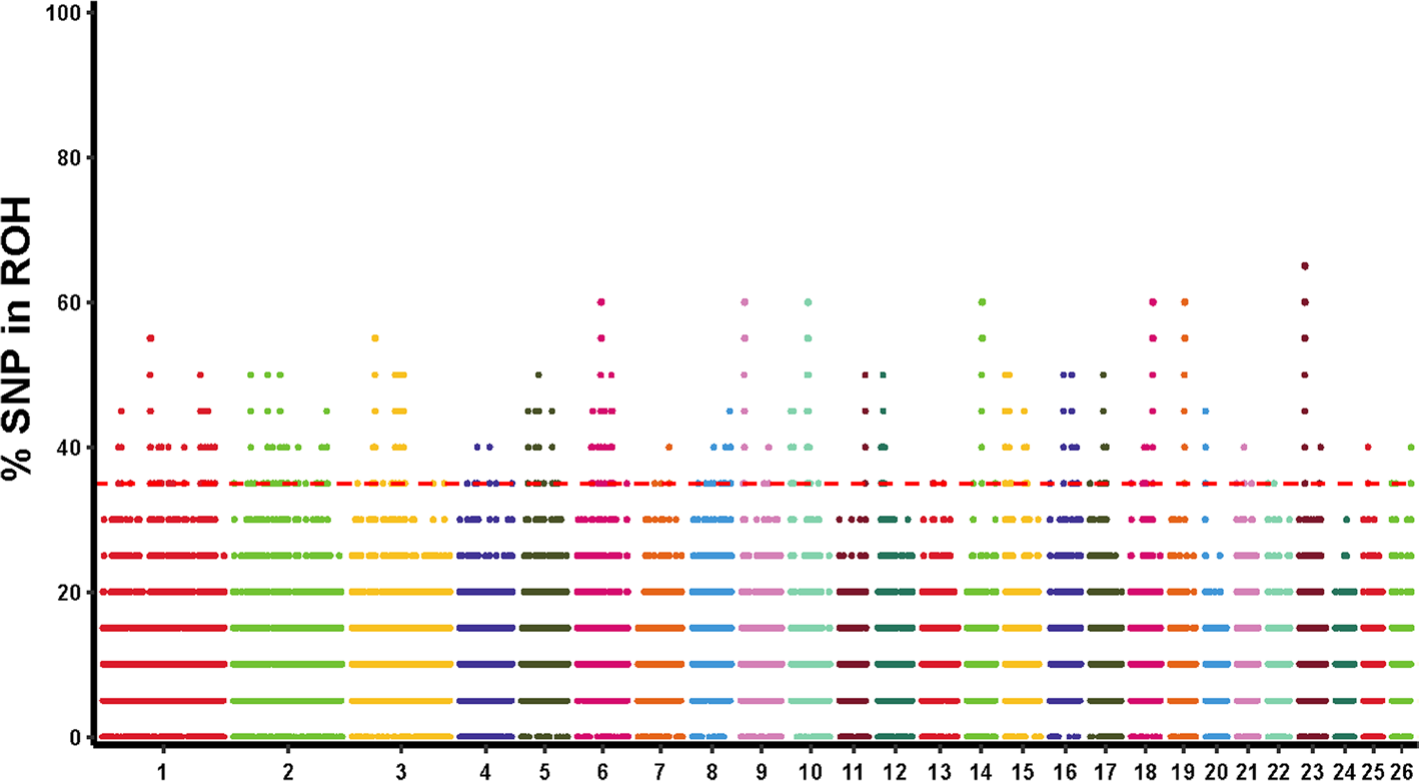
Manhattan plot of incidence of each SNP in the ROH across individuals. The abscissa represents the chromosome number of sheep and the dashed line represents the 35% threshold

With loss of nucleotide diversity and increased homozygosity, selected genomic regions tend to give rise to ROH islands compared to the rest of the genome. ROH islands are not randomly distributed across the genome, but are shared by all individuals in a population. ROH islands were evident throughout the genome, and their distribution varied in length and location on chromosomes. The most common genomic regions associated with high frequency of ROHs were detected in both sheep breeds and the percentage of SNPs was assessed by calculating the frequency of SNPs in these ROHs on autosomes, plotting a Manhattan plot (Fig. 2**)**. The maximum percentage of SNPs detected in ROH was 65%. To identify the genomic regions associated with ROH in all individuals, the top 1% of SNPs most common in ROH (occurring in more than 35% of samples) were considered candidate SNPs. A total of 66 ROH islands were obtained, as shown see Additional file 5: Table S4.

The lengths of these ROH islands ranged from 13.21 kb on OAR2 to 682.57 kb on OAR19. There are more ROH islands on OAR1 than on other autosomes. In addition, a total of 142 genes were annotated on these ROH islands (see Additional file 5: Table S4). Furthermore, the length of ROH islands was independent of the number of genes within the ROH islands. For example, the ROH islands on OAR5 are not the longest, but contain the most annotated genes, with 26. By annotating genes in each ROH island, we obtained a total of 35 shared annotated genes (see Additional file 6: Table S5). We compared these 35 shared genes with annotated genes in the total genes of ROH islands. The strongest candidate regions were found on chromosomes OAR1, OAR2, OAR6, OAR14 and OAR15 spanning multiple genes, such as *SPTA1*, *GALNTL6*, *SPP1*, *ABCG2*, *CDH1* and *PDE2A* genes (Table 2). *SPTA1* gene has been reported to be associated with red blood cell dysfunction [42], *ABCG2* gene is mainly involved in energy Metabolism-related processes [43], *PDE2A* gene is reported to be associated with lung injury and respiratory regulation [44], these three genes may be related to the adaptation to the high-altitude cold environment in Tibetan areas. In addition, *GALNTL6* [45], *SPP1* [46, 47] and *CDH1* [48] related to animal immune and developmental biological pathways, especially body weight, average daily gain, birth weight length and disease resistance QTL. This result suggests that genes related to growth and development, immune response and adaptation to hypoxia may be the selection targets for domestication and breeding improvement of Tibetan sheep in this region.

**Table 2:** The candidate genes located in genomic regions with the highest ROH frequency associated with important traits

Chr	Positions	Gene name	Gene_ID	Gene Description	Functions
1	109,570,236–109,651,920	*SPTA1*	ENSOARG00000007488	spectrin alpha chain, erythrocytic 1	Immune response
2	107,655,610–108,495,783	*GALNTL6*	ENSOARG00000015420	polypeptide N-acetylgalactosaminyltransferase-like 6 isoform X4	Body size/development
6	37,363,570–37,376,486	*SPP1*	ENSOARG00000002590	osteopontin precursor	Growth
6	37,142,499–37,274,609	*ABCG2*	ENSOARG00000001914	protein phosphatase 1 K, mitochondrial	Digestion and metabolism
14	35,614,031–35,685,913	*CDH1*	ENSOARG00000003455	cadherin-1	Disease resistance
15	50,860,329–50,950,756	*PDE2A*	ENSOARG00000005656	cGMP-dependent 3′,5′-cyclic phosphodiesterase isoform X2	Adaptive immune responses

### Genomic diversity and population structure analyses

To understand the genetic diversity of these 2 sheep breeds, we estimated expected heterozygosity (He), observed heterozygosity (He), and minor allele frequency (MAF) based on genotype frequencies (Table 3). The higher the population heterozygosity, the greater the population diversity. By comparison, we found that the OL π value (0.002751) and the Fst (0.1819) value were higher than the PO (π = 0.002653; Fst = 0.1697) value, and the value of expected heterozygosity (He) was always greater than that of observed heterozygosity (Ho) in each group. In addition, the genetic diversity of OL sheep is higher than that of PO sheep, which means that the level of genetic diversity of OL sheep is higher than that of PO sheep, and the genetic diversity of PO sheep is lower. This may be because PO sheep is a long-term artificial selection of meat quality traits. Breeds in isolated environments with low genetic diversity.

**Table 3:** Summary of genomic diversity estimates in the two sheep breeds

Group name	Sample size	Indices of genetic diversity							
		Ho	He	Fst	MAF	PLN	PPL	Na	π
OL	10	0.2267	0.2771	0.1819	0.2235	22,436,213	0.8984	1.2269	0.002751
PO	10	0.2218	0.2672	0.1697	0.2283	21,267,961	0.8517	1.2221	0.002653
Over all	20	0.2242	0.2843	0.2111	0.2046	24,972,626	1	1.2245	–

*Note*: *Ho* Observed heterozygosity, *He* Expected heterozygosity, *Fst* Fixation index, *MAF* Minor allele frequency, *PLN* Parsimony-informative sit, *PPL* Polymorphic loci, *Na* Number of observed alleles, *π* Nucleotide diversity

**Fig. 3:**
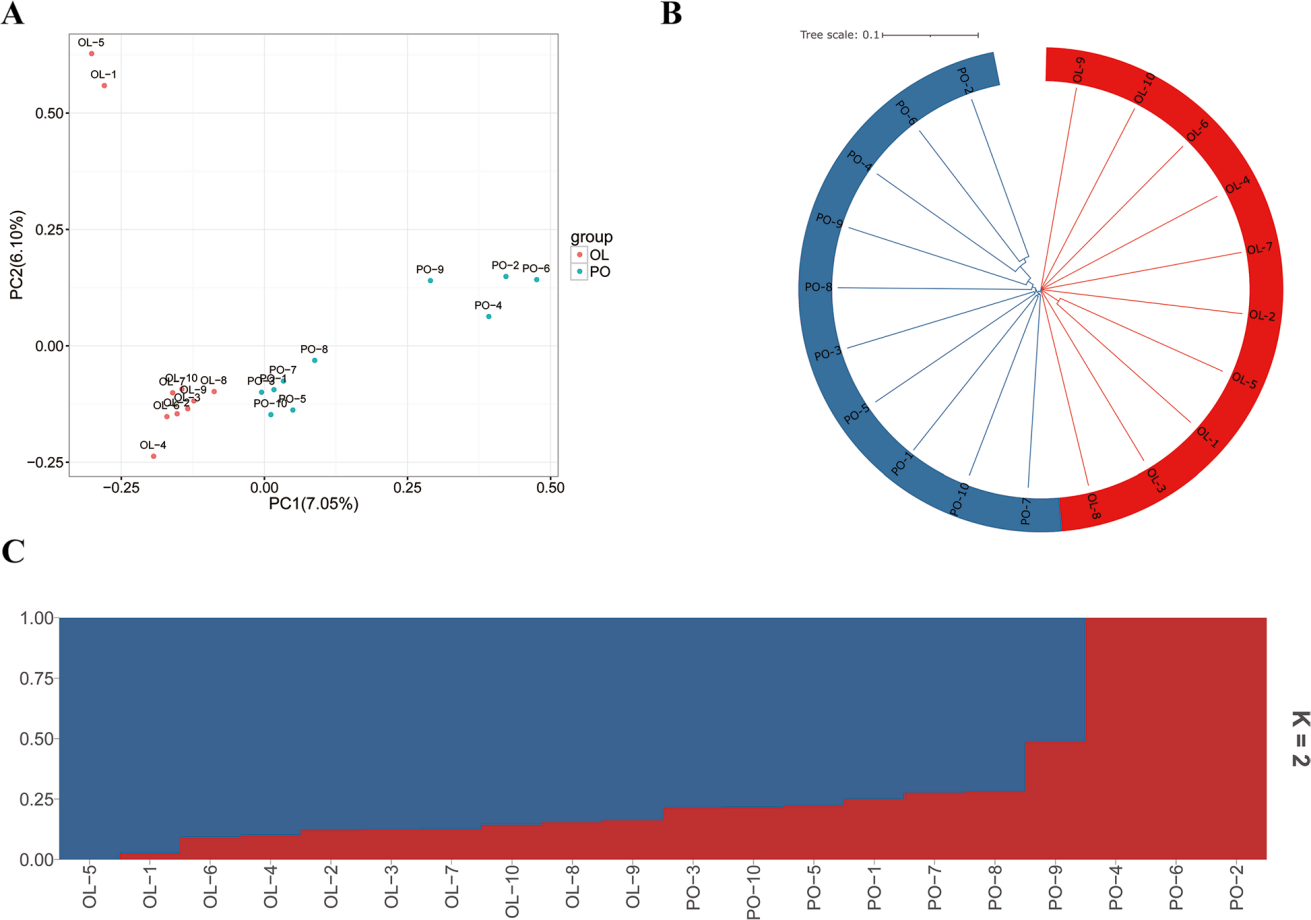
Population relationship and structural analysis of OL sheep and PO sheep varieties. **A** Principal component analysis was performed on 20 sheep. **B** Maximum likelihood tree reconstruction using RAxML and GTRGAMMA models. **C** Whole-genome mixing ratio of K = 2 in 20 sheep of two breeds

To assess the genetic relationship and structure between the two breeds, PCA, neighbor joining (NJ) tree and admixture analyses were performed. PCA results show that the first eigenvector clearly distinguished PO sheep varieties from OL sheep **(**Fig. 3A). The first two principal components explained 13.17% of the total variance and were used to visualize the relationship between the two sheep populations. In order to further study the genetic relationship of these two sheep breeds, a neighbor joining algorithm was used to construct a phylogenetic tree using the filtered SNP set.

The results of the NJ tree and admixture analysis showed that the two studied varieties were divided into different genetic groups, confirming their genetic differences. The results of the NJ tree and admixture analysis showed that the two studied varieties were divided into different genetic groups, confirming their genetic differences. Due to the geographical proximity and the low degree of differentiation of PO sheep population, there is a close kinship or gene flow between the two breeds **(**Fig. 3B**)**. To determine the proportion and/or admixture levels of common genetic ancestors, we used ADMIXTURE V 1.3.0 [34] to explore the genetic structure of the population. Combining the actual population size of our current study, and the results of the PCA analysis, we can observe two genomic clusters when K = 2. One genomic cluster was dominant in OL sheep, and two clusters were observed in PO sheep **(**Fig. 3C**)**. In addition, PO-2, PO-4, PO-6, PO-9 in the PO sheep population showed greater genetic differences than the other samples, and the genetic background of the rest of the PO sheep individuals was similar to that of the OL sheep **(**Fig. 3C**)**. The admixture analysis was consistent with the NJ results, revealing a mixed genetic composition of the Panou sheep breed. It is not difficult to see that due to the influence of living environment or artificial selection, there is differentiation phenomenon in Panou sheep.

### Genetic signature of positive selection in Panou sheep

In domesticated species, an understanding of phenotypic diversity among varieties or species can often be used in conjunction with selection elimination analysis to better understand the underlying biological significance of controlling phenotypic variation. Population evolutionary selection elimination analysis means that the elimination of nucleotide polymorphisms in a region of the genome as a result of selection. It was the imprint that left by positive selection on the genome of a species [6]. Compared with the control population, the genetic diversity of the selected population was significantly reduced in the regions where selection elimination occurred, which is typical of domesticated regions. In order to better understand the underlying genetic rules of phenotypic traits, production traits and adaptive traits between the two sheep breeds, OL sheep were used as the control group and PO sheep as the selection group, and two methods of F_st_ and π ratio were used to identify the selection characteristics. The results of the selection elimination analysis of Fst and π ratio (see Additional file 7: Figs. S2 and S3) showed that they jointly screened for a strong selection signal, which facilitated the selection of target genes.

**Fig. 4:**
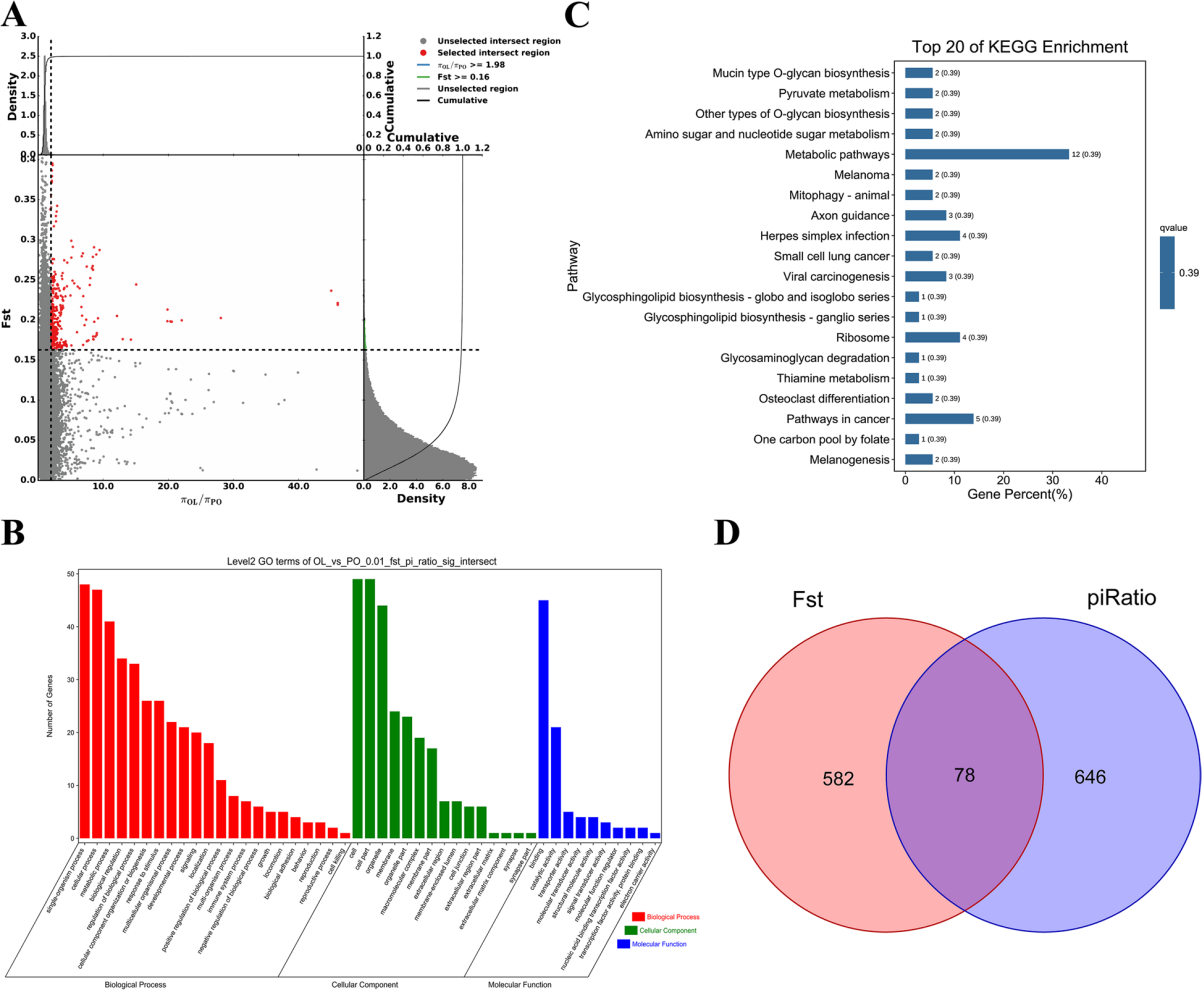
Analysis of the signatures of positive selection in the genome of samples. Genomic landscape of the Fst values and π ratio values. **A** Using Fst and π ratio methods to determine the genomic Manhattan selection feature map and global map of selection signals. **B**&**C** GO and KEGG heatmap selected analyzed by Fst and π ratio. The top 1% was chosen as the significant threshold for Fst and π ratio. **D** The venn map of selected genes combining Fst and π ratio with the parameters determined by top1%

According to the results of Fst and π ratio, significant regions were screened by top1%, and genes in these selected regions were identified (Fig. 4A). A total of 78 genes were identified in the candidate regions of the 2 breeds (Fig. 4D, see Additional file 8: Table S6). We performed Gene Ontology analysis (Fig. 4B) and Kyoto Encyclopedia of Genes and Genomes (Fig. 4C) analysis of the common genes of the two breeds by individual selection method.

The results of enrichment analysis showed that 78 common genes were enriched in 46 biological processes, 17 molecular functions and 14 cellular components in Gene Ontology. We screened genes enriched in the top 20 GO and KEGG with differences in enrichment scores as candidate genes. In GO terms, it is related to multiple molecular functions, cellular components, and biological processes involved, such as: ligase activity, formation of aminoacyl tRNA and related compounds (GO:0004812; *P < 0.01*), AP-type membrane-coated adapter complex (GO:0030119; *P < 0.05*), canonical Wnt signaling pathway (GO:0060070; *P < 0.01*), etc. (see Additional file 9: Table S7). The top three significantly enriched important pathways in KEGG are related to mucin-type O-glycan biosynthesis (ko00512; *P < 0.01*), pyruvate metabolism (ko00620; *P < 0.05*), other types of O-glycan biosynthesis (ko00514; *P < 0.05*) correlation (see Additional file 10: Table S8). Finally, 7 candidate genes related to hypoxia response, growth and development, disease resistance, coat color and reproductive traits were obtained, namely *VAT1*, *IFI35*, *HDAC9*, *MITF*, *TCHHL1*, *AOC3* and *PTK2* (Table 4).

**Table 4:** Candidate genes detected by the top 1% Fst and π ratio values

Chr	Symbol	Ensembl Gene ID	Description
1	*TCHHL1*	ENSOARG00000021082	trichohyalin-like protein 1 [*Ovis aries*]
4	*HDAC9*	ENSOARG00000009641	PREDICTED: histone deacetylase 9 isoform X1 [*Bos indicus*]
9	*PTK2*	ENSOARG00000003418	focal adhesion kinase 1 isoform X18 [*Ovis aries*]
11	*AOC3*	ENSOARG00000000273	primary amine oxidase, lung isozyme [*Ovis aries*]
11	*IFI35*	ENSOARG00000004413	interferon-induced 35 kDa protein isoform X1 [*Ovis aries*]
11	*VAT1*	ENSOARG00000004544	synaptic vesicle membrane protein VAT-1 homolog [*Ovis aries*]
19	*MITF*	ENSOARG00000009833	microphthalmia-associated transcription factor isoform X1 [*Ovis aries*]

## Discussion

In the context of animal breed differentiation, the selection characteristics of populations are very important. The detection of selection traits in different populations can not only reveal the mechanism of current artificial selection breeding, but also provide new insights into phenotypic variation of new varieties and the search for genes associated with important traits. This study provides useful information for the sustainable utilization and conservation of Tibetan sheep genetic resources, and also provides a deeper understanding of genomic diversity and variation information related to Tibetan sheep adaptation and production traits.

### Genome diversity

Assessing genetic variability within breeds or populations can provide insights into designing breeding improvement strategies for indigenous sheep genetic resources [49, 50]. In general, the diversity of all sheep with a high degree of selection and breeding is relatively low. Breeds with a low diversity show a high coverage by runs of homozygosity [51]. The genomic diversity of Oula sheep and Panou sheep in this study was higher than Suffolk sheep (π = 0.002467) and Dorper sheep (π = 0.002526), but lower than Asiatic mouflon, and urial (π = 0.0032–0.0044) [14]. There were some differences in nucleotide diversity from different breeds. Substantially, the values of nucleotide diversity calculated here are in the range of earlier estimates in domestic sheep (π = 2.44–2.84) [52]. By comparison, we found that the genetic diversity of OL sheep had higher values of each parameter than PO, and combined with ROH length and LD decay, the genetic diversity of OL sheep was higher than that of PO sheep. The F_ROH_ results showed that the inbreeding coefficient of OL sheep was lower, indicating that the decline of inbred lines was not serious. Since PO sheep have the higher inbreeding coefficient, explicit measures should be taken to prevent the decline of inbred lines. The relative effective population size was estimated as OL > PO, and the genetic diversity determined by the effective population size was consistent with the ROH and LD decay results.

ROH estimation is a useful tool for exploring population genetic diversity, providing statistical evaluation of population history information and predicting underlying genomic structure [53]. ROH can be used to identify regions that adversely affect phenotype when they are homozygous, and can also be used to detect associations between economic traits and genes in these regions [54]. In addition, the effects of selection pressure and breeding management on sheep genomes may imprint on ROH length and some ROH length categories can serve as indicators of pedigree and breed group history. In our study, the ROH length categories of the two groups are mainly concentrated in 0-1Mb. As described by Purfield et al. [55], a relatively short ROH is the most possibly related to ancestral genetics or potential bottlenecks, while longer ROH is more likely related to relatively recent inbreeding. Thus, because of strong LDs, typically extending to about 100 Kb, short ROHs are common throughout the sheep genome [56]. In addition, the ROH level of PO sheep was higher than that of OL sheep and Poll Dorset sheep [9], but lower than that of Yabuyi, Karakul and Wadi sheep [57]. It shows that PO sheep have relatively high genetic diversity, relatively low population inbreeding level, proper breeding management and adequate population size, which are conducive to better adaptation of this breed to the long-term growth environment.

### Population structure

The PCA, NJ phylogenetic trees and admixture analyses revealed a common clustering pattern. The PCA analysis showed that the first and second principal components explained 13.17% of the total variance for visualizing the relationship of the two sheep populations. The results indicated that there was a certain genetic differentiation between PO and OL. In addition, the distribution of PO was more dispersed than that of OL, reflecting the greater genetic variation among PO individuals.

The results of LD analysis showed that the average r^2^ value of PO sheep decreased rapidly with increasing genomic distance and remained unchanged after 100–200 Kb. In the first 50 Kb, r^2^ has the fastest drop. The reason for this phenomenon may be that PO sheep are subjected to a certain intensity of selection during the breeding process, resulting in a decrease in the genetic diversity of the population and an increase in the correlation (linkage) between loci. This result is consistent with Zhang et al. [9]. Therefore, extensive mating among close relatives may be responsible for the high proportion of fixed alleles, resulting in low genetic diversity in PO sheep. However, the inbreeding level of these two populations is limited and much lower than that of Dorper sheep [58]. It may be because the Dorper sheep is a specialized breed, and the breed formation process is constantly artificially selected in the direction of meat traits. Overall, the OL sheep and PO sheep breeds are well managed and the effective population size is sufficiently large.

### Selection characteristics of candidate genes

Genome-wide sequence analysis revealed genes associated with different origins and domestication in sheep European and Asian Europe and Asia. It was found that the splitting time between Argali and domestic sheep at ~ 0.12–0.15 Mya was closer than previous estimates [14]. Li et al. deciphered the genetic basis concerning the domestication of unique phenotypes in sheep by studying the whole genomes of wild sheep, local sheep breeds and improved sheep breeds. And the genomic diversity of local sheep breeds was found to be largely preserved in the improved breeds, consistent with previous evidence that metabolic processes and olfactory transduction were the main functional categories selected for during sheep domestication [7, 59]. A strong signature of adaptive infiltration from Argali to Tibetan sheep was detected, with infiltrating genes involved in hypoxic and UV signaling pathways (e.g. *HBB* and *MITF*) and associated with morphological features such as horn size and shape (e.g. *RXFP2*). Gene infiltration was associated with adaptation to the extreme environment of the Tibetan Plateau [4]. Theoretically, the specific functions of these domestication-associated candidate genes suggest that the traits have been targets of intense selection pressure during domestication, ultimately leading to typical morphological, production, physiological and behavioral differences between domestic sheep and wild sheep [60].

It is known that detection of common selection signatures by more than one method can provide stronger evidence for selection of specific genomic regions. Various statistical tools, such as Fst and π ratio, are recognized tools in the identification of livestock selection traits because they can point to the direction of selection required to identify a range of new regions as potential selection targets. Using Fst analysis, Zhang et al. examined genome-wide selection signals in five sheep breeds and identified genes associated with important traits. For example, *RXFP2*, *GHR* and *ASIP* were associated with horn shape, growth and lipid metabolism [12]. Plateau adaptations is a complex trait regulated by several factors, such as hypoxia-inducible factors, angiogenesis, vasodilation and glycolytic metabolism [5]. We also found 20 significantly over-represented GO categories (binomial distribution test, *P < 0.05*) for PO sheep (see Additional file 9: Table S7**)**. The GO clusters were primarily associated with acetylgalactosaminyltransferase activity，pigment cell differentiation，canonical Wnt signaling pathway. These functional clusters are biologically relevant to the plateau adaptations because they are involved in energy metabolism, oxidative responses and stress responses, all of which are important regulators of the animal’s response to the high altitude, low oxygen environment. We found two positively selected genes (*GALNT16* and *C1GALT1C1*) regulating the acetylgalactosaminyltransferase activity, two genes (*MITF* and *AP1G2*) affecting the pigment cell differentiation, one gene (*MITF*) influencing the canonical Wnt signaling pathway. And also, *MITF* and *GALNT16* were significantly enriched in the KEGG pathway of melanoma and mucin type O-glycan biosynthesis, respectively **(**Fig. 4C**)**. In addition, 7 candidate genes (*VAT1、IFI35、HDAC9、MITF、TCHHL1、AOC3 and PTK2*) provide additional evidence for the evolution of selection in the adaptation of PO sheep to the plateau environment.

Tibetan sheep are exposed to strong ultraviolet radiation for a long time, which will inevitably cause some damage to their own skin and organism health. Previous studies have found that vesicular amine transporter 1 (*VAT1*) [61], interferon-inducible protein 35 (*IFI35*) [62], and amine oxidase, copper containing 3 (*AOC3*) [63] were associated with immune response and disease resistance in animal organisms. Microphthalmia-associated transcription factor (*MITF*) is a basic helix-loop-helix-leucine zipper transcription factor that regulates melanocyte differentiation and development and pigment cell-specific transcription of melanogenesis enzyme genes [64]. Cheli et al. found that *MITF* transcriptionally activates HIF-1ɑ and thus promotes tumor angiogenesis. Hypoxia inducible factor-1ɑ (HIF-1ɑ) has not only anti-apoptotic effects in melanoma, but also stimulates the production of vascular endothelial growth factor (VEGF) [65]. *MITF* gene could be regulated by G protein coupled receptor 143 (GPR143) and soluble guanylate cyclase (SGC), and participate in the formation of sheep coat color [66]. The black fur of Tibetan sheep is related to the high expression of *MITF* in hair follicles, and *MITF* may play an important role in the formation of Tibetan sheep fur color [67].

Studies had shown that ultraviolet radiation will produce a large number of free radicals in the skin, which will lead to the peroxidation of the cell membrane, so that the melanocytes will produce more melanin, which will be distributed to the stratum corneum of the epidermis, causing black spots. The study found that the expression of *TCHHL1* gene was enhanced in UV-irradiated-damaged epidermal proliferating keratinocytes [68]. These findings suggest that *TCHHL1* is involved in the proliferation of keratinocytes, and that *TCHHL1* plays an important role in the homeostasis of normal epidermis, reducing damage to basal cells and squamous cells of skin hair follicles caused by ultraviolet rays [69]. It can be seen that during the selection and breeding of PO sheep, there had been a conscious effort to enhance the plateau adaptation characteristics of Tibetan sheep through selection.

Generally speaking, Tibetan sheep are small individuals and belong to low lambing sheep breed with seasonal estrus [70]. Nevertheless, people still hope to improve the lambing rate and improve the body size or meat quality of Tibetan sheep through purposeful artificial selection breeding. Several reports suggest that protein tyrosine kinases (PTKs) are important for regulating intracellular events following stimulation of granulosa cells with various factors. Phosphorylation of intracellular signaling molecules by PTK is essential for regulating the dynamics of follicle growth and atresia [71], locally produced survival factors, epidermal growth factor (EGF) and basic fibroblast growth factor (bFGF), these receptors have a PTK domain in their molecular structure and inhibit apoptosis as effectively as gonadotropins [72]. Not only that, focal adhesion kinase (FAK/PTK2) interacts with many signaling partners and is involved in cell adhesion, survival and other important biological processes [73]. The study by Boruah et al. also confirmed that *PTK2* gene may be involved in the development of follicles and ovaries in the final stage of Hu sheep [74]. Meat quality is a quantitative trait regulated by complex factors such as glycolysis, cell cycle, proteolysis, protein ubiquitination and apoptosis. Some studies have found that histone deacetylase 9 (*HDAC9*) is related to the development of muscle structure in sheep [75]. *HDAC9* is strongly expressed in skeletal muscle during embryogenesis but is downregulated in skeletal muscle after birth [76]. Previous studies have found that *HDAC9* weakens the transcriptional loop of muscle differentiation through a negative feedback loop by inhibiting the transcriptional activity of *MEF2* [77]. Furthermore, in studying muscle differentiation in vitro, *HDAC9* expression initially increased but then decreased to basal levels [78]. These findings reveal the molecular basis for the robustness of muscle phenotype and fine-tune muscle gene expression by regulating the expression and activity of *HDAC9*, a key negative regulator of the muscle gene program.

The above results suggest that these genes may play a dominant role in the regulation of immune response, disease resistance, fecundity, growth and developmental body size of PO sheep. Thus, the complex genetic mechanism of artificial selection for breeding Tibetan sheep in the highland environment is further demonstrated.

## Conclusion

This study provides a comprehensive understanding of the phylogenetic relationship between PO sheep and OL sheep. PCA, Structure and NJ-tree analysis showed that PO sheep is different from OL sheep. After long-term artificial selection and breeding, this breed has gradually formed a unique gene pool that is different from other Tibetan sheep. Strong selection by Fst and π ratio enriched canonical Wnt signaling pathway (GO:0060070) and Melanoma (ko05218), identifying *MITF*, a common gene adapted to ultraviolet radiation in the plateau. And the other identified candidate genes in PO sheep associated with hypoxia adaptation, growth and development, disease resistance and reproductive traits, providing opportunities to further explore the genetic diversity and genetic basis behind different phenotypes in Tibetan sheep, as well as providing valuable information for future studies on genotype-phenotype relationships and improved breeding in PO sheep.

## Supplementary Information

**Additional file 1: Table S1.** Sampling information of Tibetan sheep breeds (*Ovis aries*).

**Additional file 2: Table S2.** Numbers and distribution of SNPs-InDel in the resequenced sheep breeds.

**Additional file 3: Table S3.** The numbers and distribution of SNPs-InDel in the exons.

**Additional file 4: Figure S1.** Pairwise sequential Markovian coalescent (PSMC) analysis results for the Tibetan sheep inferred variations in Ne over the last 10^6^ years.

**Additional file 5: Table S4.** Distribution of ROH islands throughout the genome.

**Additional file 6: Table S5.** Distribution and annotation information of 35 shared genes in ROH island.

**Additional file 7: Figure S2.** Analysis of the signatures of positive selection in the genome of samples and genomic landscape of the Fst values. **Figure S3.** Analysis of the signatures of positive selection in the genome of samples and genomic landscape of the π ratio values.

**Additional file 8: Table S6.** The list of 78 genes under selection across the two breeds.

**Additional file 9: Table S7.** GO top 20:GO enrichment analysis for candidate genes as determined by top 1% Fst and π ratio value methods (*p < 0.05*).

**Additional file 10: Table S8.** KEGG top 20: KEGG enrichment analysis for candidate genes as determined by top 1% Fst and π ratio value methods (*p < 0.05*).

## Data Availability

The datasets generated in this paper can be found at Sequence Read Archive: PRJNA797957.

## References

[1] Zhao E, Yu Q, Zhang N, Kong D, Zhao Y. Mitochondrial DNA diversity and the origin of Chinese indigenous sheep. Trop Anim Health Prod. 2013;45(8):1715-22. 10.1007/s11250-013-0420-5 23709123

[2] Liu J, Ding X, Zeng Y, Yue Y, Guo X, Guo T, Chu M, Wang F, Han J, Feng R (2016). Genetic diversity and phylogenetic evolution of Tibetan sheep based on mtDNA D-loop sequences. PLoS One.

[3] Chessa B, Pereira F, Arnaud F, Amorim A, Goyache F, Mainland I, et al. Revealing the history of sheep domestication using retrovirus integrations. Science (New York, NY). 2009;324(5926):532–6. 10.1126/science.1170587 PMC3145132 19390051

[4] Hu XJ, Yang J, Xie XL, Lv FH, Cao YH, Li WR, Liu MJ, Wang YT, Li JQ (2019). The genome landscape of Tibetan sheep reveals adaptive introgression from argali and the history of early human settlements on the Qinghai-Tibetan plateau. Mol Biol Evol.

[5] Yang J, Li W, Lv F, He S, Tian S, Peng W, et al. Whole-genome sequencing of native sheep provides insights into rapid adaptations to extreme environments. Mol Biol Evol. 2016;33(10):2576–92. 10.1093/molbev/msw129 PMC5026255 27401233

[6] Nosrati M, Asadollahpour Nanaei H, Amiri Ghanatsaman Z, Esmailizadeh A. Whole genome sequence analysis to detect signatures of positive selection for high fecundity in sheep. Reprod Domest Anim. 2019;54(2):358–64. 10.1111/rda.13368 30359467

[7] Li X, Yang J, Shen M, Xie X, Liu G, Xu Y, Lv F, Yang H, Yang Y, Liu C (2020). Whole-genome resequencing of wild and domestic sheep identifies genes associated with morphological and agronomic traits. Nat Commun.

[8] Cao Y, Xu S, Shen M, Chen Z, Gao L, Lv F, et al. Historical introgression from wild relatives enhanced climatic adaptation and resistance to pneumonia in sheep. Mol Biol Evol. 2021;38(3):838–55. 10.1093/molbev/msaa236 PMC7947771 32941615

[9] Zhang Y, Xue X, Liu Y, Abied A, Ding Y, Zhao S, Wang W, Ma L, Guo J, Guan W (2021). Genome-wide comparative analyses reveal selection signatures underlying adaptation and production in Tibetan and poll Dorset sheep. Sci Rep.

[10] Liu JB, Guo J, Wang F, Yue YJ, Zhang WL, Feng RL, et al. Carcass and meat quality characteristics of Oula lambs in China. Small Rumin Res. 2015;123(2–3):251–9.

[11] Guo X, Liu JB, Zeng YF, Ding XZ, Bao PJ, Yan P, Pei J. Study on complete mitochondrial genome of Oula sheep (Ovis aries). Agric Sci Technol. 2017;18(08):1365-66.

[12] Zhang L, Mousel M, Wu X, Michal J, Zhou X, Ding B, Dodson M, El-Halawany N, Lewis G, Jiang Z (2013). Genome-wide genetic diversity and differentially selected regions among Suffolk, Rambouillet, Columbia, Polypay, and Targhee sheep. PLoS One.

[13] Akey J, Zhang G, Zhang K, Jin L, Shriver M. Interrogating a high-density SNP map for signatures of natural selection. Genome Res. 2002;12(12):1805–14. 10.1101/gr.631202 PMC187574 12466284

[14] Chen Z, Xu Y, Xie X, Wang D, Aguilar-Gómez D, Liu G, Li X, Esmailizadeh A, Rezaei V, Kantanen J (2021). Whole-genome sequence analysis unveils different origins of European and Asiatic mouflon and domestication-related genes in sheep. Commun Biol.

[15] Alshawi A, Essa A, Al-Bayatti S, Hanotte O (2019). Genome analysis reveals genetic admixture and signature of selection for productivity and environmental traits in Iraqi cattle. Front Genet.

[16] Bickhart DM, Hou Y, Schroeder SG, Alkan C, Cardone MF, Matukumalli LK, Song J, Schnabel RD, Ventura M, Taylor JF (2012). Copy number variation of individual cattle genomes using next-generation sequencing. Genome Res.

[17] Zhangyuan P, Xiaoyun H, Xiangyu W, Xiaofei G, Mingxing C (2016). Selection signature in domesticated animals. Hereditas.

[18] Naval-Sánchez M, Porto-Neto L, Cardoso D, Hayes B, Daetwyler H, Kijas J, Reverter A (2020). Selection signatures in tropical cattle are enriched for promoter and coding regions and reveal missense mutations in the damage response gene HELB. Genet Sel Evol.

[19] Ai H, Yang B, Li J, Xie X, Chen H, Ren J (2014). Population history and genomic signatures for high-altitude adaptation in Tibetan pigs. BMC Genomics.

[20] Wang M, Li Y, Peng M, Zhong L, Wang Z, Li Q, et al. Genomic analyses reveal potential independent adaptation to high altitude in Tibetan chickens. Mol Biol Evol. 2015;32(7):1880–9. 10.1093/molbev/msv071 25788450

[21] Wei C, Wang H, Liu G, Zhao F, Kijas J, Ma Y, Lu J, Zhang L, Cao J, Wu M (2016). Genome-wide analysis reveals adaptation to high altitudes in Tibetan sheep. Sci Rep.

[22] Bertolini F, Servin B, Talenti A, Rochat E, Kim E, Oget C, Palhière I, Crisà A, Catillo G, Steri R (2018). Signatures of selection and environmental adaptation across the goat genome post-domestication. Genet Sel Evol.

[23] Hornyak T, Jiang S, Guzmán E, Scissors B, Tuchinda C, He H, et al. Mitf dosage as a primary determinant of melanocyte survival after ultraviolet irradiation. Pigment Cell Melanoma Res. 2009;22(3):307–18. 10.1111/j.1755-148X.2009.00551.x PMC6980044 19192212

[24] Megdiche S, Mastrangelo S, Hamouda MB, Lenstra JA, Ciani E. Merino and merino-derived sheep breeds: a further look at genome-wide selection signatures for wool traits. Front Genet. 2019;10:1025–39. 10.3389/fgene.2019.01025 PMC6824410 31708969

[25] Li H, Durbin R. Fast and accurate short read alignment with burrows-wheeler transform. Bioinformatics (Oxford, England). 2009;25(14):1754–60. 10.1093/bioinformatics/btp324 PMC2705234 19451168

[26] McKenna A, Hanna M, Banks E, Sivachenko A, Cibulskis K, Kernytsky A, et al. The genome analysis toolkit: a MapReduce framework for analyzing next-generation DNA sequencing data. Genome Res. 2010;20(9):1297–303. 10.1101/gr.107524.110 PMC2928508 20644199

[27] Petr D, Adam A, Goncalo A, Albers CA, Eric B. The variant call format and VCFtools. Bioinformatics (Oxford, England). 2011;27(15):2156–8. 10.1093/bioinformatics/btr330 PMC3137218 21653522

[28] Purcell S, Neale B, Todd-Brown K, Thomas L, Ferreira M, Bender D, et al. PLINK: a tool set for whole-genome association and population-based linkage analyses. Am J Hum Genet. 2007;81(3):559–75. 10.1086/519795 PMC1950838 17701901

[29] Kai W, Li M, Hakon H (2010). ANNOVAR: functional annotation of genetic variants from high-throughput sequencing data. Nucleic Acids Res.

[30] Mastrangelo S, Ciani E, Sardina M, Sottile G, Pilla F, Portolano B (2018). Runs of homozygosity reveal genome-wide autozygosity in Italian sheep breeds. Anim Genet.

[31] Mastrangelo S, Sardina M, Tolone M, Di Gerlando R, Sutera A, Fontanesi L, et al. Genome-wide identification of runs of homozygosity islands and associated genes in local dairy cattle breeds. Animal. 2018;12(12):2480–8. 10.1017/S1751731118000629 29576040

[32] Terhorst J, Kamm J, Song Y. Robust and scalable inference of population history from hundreds of unphased whole genomes. Nat Genet. 2017;49(2):303–9. 10.1038/ng.3748 PMC5470542 28024154

[33] Barrett J, Fry B, Maller J, Daly M. Haploview: analysis and visualization of LD and haplotype maps. Bioinformatics (Oxford, England). 2005;21(2):263–5. 10.1093/bioinformatics/bth457 15297300

[34] Tang H, Peng J, Wang P, Risch N (2005). Estimation of individual admixture: analytical and study design considerations. Genet Epidemiol.

[35] Paradis E, Claude J, Strimmer K. APE: analyses of Phylogenetics and evolution in R language. Bioinforma. 2004;20(2):289–90. 10.1093/bioinformatics/btg412 14734327

[36] Pfeifer B, Wittelsbürger U, Onsins S, Lercher MJ. PopGenome: an efficient Swiss Army knife for population genomic analyses in R. Mol Biol Evol. 2014;31(7):1929–36. 10.1093/molbev/msu136 PMC4069620 24739305

[37] Gallone B, Steensels J, Prahl T, Soriaga L, Saels V, Herrera-Malaver B, Merlevede A, Roncoroni M, Voordeckers K, Miraglia L (2016). Domestication and divergence of Saccharomyces cerevisiae beer yeasts. Cell.

[38] Hudson R, Slatkin M, Maddison W. Estimation of levels of gene flow from DNA sequence data. Genet. 1992;132(2):583–9. 10.1093/genetics/132.2.583 PMC1205159 1427045

[39] Nei M, Li W. Mathematical model for studying genetic variation in terms of restriction endonucleases. Proc Natl Acad Sci U S A. 1979;76(10):5269–73. 10.1073/pnas.76.10.5269 PMC413122 291943

[40] Tajima F. Statistical method for testing the neutral mutation hypothesis by DNA polymorphism. Genet. 1989;123(3):585–95. 10.1093/genetics/123.3.585 PMC1203831 2513255

[41] Lin T, Zhu G, Zhang J, Xu X, Yu Q, Zheng Z, et al. Genomic analyses provide insights into the history of tomato breeding. Nat Genet. 2014;46(11):1220–6. 10.1038/ng.3117 25305757

[42] Andolfo I, Martone S, Rosato B, Marra R, Gambale A, Forni G, Pinto V, Göransson M, Papadopoulou V, Gavillet M (2021). Complex modes of inheritance in hereditary red blood cell disorders: a case series study of 155 patients. Genes.

[43] Hou Y, Li C, Palaniyandi K, Magtibay P, Homolya L, Sarkadi B, et al. Effects of putative catalytic base mutation E211Q on ABCG2-mediated methotrexate transport. Biochem. 2009;48(38):9122–31. 10.1021/bi900675v PMC2770347 19691360

[44] Schmidt E, Damarla M, Rentsendorj O, Servinsky L, Zhu B, Moldobaeva A, et al. Soluble guanylyl cyclase contributes to ventilator-induced lung injury in mice. Am J Phys Lung Cell Mol Phys. 2008;295(6):L1056–65. 10.1152/ajplung.90329.2008 PMC2604795 18849438

[45] Seifi Moroudi R, Ansari Mahyari S, Vaez Torshizi R, Lanjanian H, Masoudi-Nejad A. Identification of new genes and quantitative trait locis associated with growth curve parameters in F2 chicken population using genome-wide association study. Anim Genet. 2021;52(2):171–84. 10.1111/age.13038 33428266

[46] Lim W, Jeong W, Kim J, Ka H, Bazer F, Han J, Song G (2012). Differential expression of secreted phosphoprotein 1 in response to estradiol-17β and in ovarian tumors in chickens. Biochem Biophys Res Commun.

[47] La Y, Zhang X, Li F, Zhang D, Wang W (2019). Molecular characterization and expression of SPP1, LAP3 and LCORL and their association with growth traits in sheep. Genes.

[48] Roy FV, Berx G. The cell-cell adhesion molecule E-cadherin. Cell Mol Life Sci. 2008;65(23):3756–88. 10.1007/s00018-008-8281-1 PMC11131785 18726070

[49] Getachew T, Haile A, Mészáros G, Rischkowsky B, Slkner J (2019). Genetic diversity, population structure and runs of homozygosity in Ethiopian short fat-tailed and Awassi sheep breeds using genome-wide 50k SNP markers. Livest Sci.

[50] Wei C, Wang H, Liu G, Wu M, Cao J, Liu Z, Liu R, Zhao F, Zhang L, Lu J (2015). Genome-wide analysis reveals population structure and selection in Chinese indigenous sheep breeds. BMC Genomics.

[51] Lv F, Cao Y, Liu G, Luo L, Lu R, Liu M, Li W, Zhou P, Wang X, Shen M (2022). Whole-genome resequencing of worldwide wild and domestic sheep elucidates genetic diversity, introgression, and Agronomically important loci. Mol Biol Evol.

[52] Pan Z, Li S, Liu Q, Wang Z, Zhou Z, Di R, Miao B, Hu W, Wang X, Hu X (2018). Whole-genome sequences of 89 Chinese sheep suggest role of RXFP2 in the development of unique horn phenotype as response to semi-feralization. GigaScience.

[53] Bjelland DW, Weigel KA, Vukasinovic N, Nkrumah JD (2013). Evaluation of inbreeding depression in Holstein cattle using whole-genome SNP markers and alternative measures of genomic inbreeding. J Dairy Sci.

[54] Szmato AT, Gurgul A, Ropka-Molik K, Jasielczuk I, ZaBek T, Bugno-Poniewierska M. Characteristics of runs of homozygosity in selected cattle breeds maintained in Poland. Livest Sci. 2016;188:72–80.

[55] Purfield D, Berry D, McParland S, Bradley D (2012). Runs of homozygosity and population history in cattle. BMC Genet.

[56] Mastrangelo S, Di Gerlando R, Tolone M, Tortorici L, Sardina M, Portolano B (2014). Genome wide linkage disequilibrium and genetic structure in Sicilian dairy sheep breeds. BMC Genet.

[57] Abied A, Xu L, Sahlu BW, Xing F, Ma Y (2020). Genome-wide analysis revealed homozygosity and demographic history of five Chinese sheep breeds adapted to different environments. Genes.

[58] Zhao F, Wang G, Zeng T, Wei C, Zhang L, Wang H, et al. Estimations of genomic linkage disequilibrium and effective population sizes in three sheep populations. Livest Sci. 2014;170:22–9.

[59] Naval-Sanchez M, Nguyen Q, McWilliam S, Porto-Neto L, Tellam R, Vuocolo T, Reverter A, Perez-Enciso M, Brauning R, Clarke S (2018). Sheep genome functional annotation reveals proximal regulatory elements contributed to the evolution of modern breeds. Nat Commun.

[60] Hunter P. The genetics of domestication: research into the domestication of livestock and companion animals sheds light both on their "evolution" and human history. EMBO Rep. 2018;19(2):201–5. 10.15252/embr.201745664 PMC5797965 29335247

[61] Yang P, Wang K, Zhang C, Wang Z, Liu Q, Wang J, et al. Novel roles of VAT1 expression in the immunosuppressive action of diffuse gliomas. Cancer Immunol Immunother. 2021;70(9):2589–600. 10.1007/s00262-021-02865-z PMC10992787 33576871

[62] MiR-146b-5p targets IFI35 to inhibit inflammatory response and apoptosis via JAK1/STAT1 signalling in lipopolysaccharide-induced glomerular cells. Autoimmunity 2021, 54(7):430–438. 10.1080/08916934.2020.1864730 34435525

[63] Dunkel J, Aguilar-Pimentel JA, Ollert M, Fuchs H, Gailus-Durner V, Angelis M, et al. Endothelial amine oxidase AOC3 transiently contributes to adaptive immune responses in the airways. Eur J Immunol. 2014;44(11):3232-9. 10.1002/eji.201444563 25116373

[64] Saravanaperumal SA, LaTerza R, Pediconi D. Alternative splicing of the sheep MITF gene: novel transcripts detectable in skin. Gene. 2014;552(1):165–75. 10.1016/j.gene.2014.09.031 25239663

[65] Cheli Y, Giuliano S, Fenouille N, Allegra M, Hofman V, Hofman P, et al. Hypoxia and MITF control metastatic behaviour in mouse and human melanoma cells. Oncogene. 2012;31(19):2461–70. 10.1038/onc.2011.425 21996743

[66] Yang S, Zhang J, Ji K, Jiao D, Fan R, Dong C. Characterization and expression of soluble guanylate cyclase in skins and melanocytes of sheep. Acta Histochem. 2016;118(3):219–24. 10.1016/j.acthis.2016.01.002 26805580

[67] Han JL, Yang M, Guo TT, Yue YJ, Liu JB, Niu CE, Wang CF, Yang BH (2015). Molecular characterization of two candidate genes associated with coat color in Tibetan sheep (Ovis arise). Agric Sci China.

[68] Makino T, Mizawa M, Yoshihisa Y, Shimizu T (2017). 136 the expression of trichohyalin-like 1 protein in human skin xenotransplants is enhanced by ultraviolet B irradiation. J Investig Dermatol.

[69] Makino T, Mizawa M, Yoshihisa Y, Yamamoto S, Shimizu T (2020). Trichohyalin-like 1 protein plays a crucial role in proliferation and anti-apoptosis of normal human keratinocytes and squamous cell carcinoma cells. Cell Death Dis.

[70] Du L-X (2011). Animal genetic resources in China.

[71] Chun SY, Eisenhauer KM, Minami S, Billig H, Perlas E, Hsueh AJ. Hormonal regulation of apoptosis in early antral follicles: follicle-stimulating hormone as a major survival factor. Endocrinol. 1996;4:1447–56. 10.1210/endo.137.4.8625923 8625923

[72] Chun SY, Billig H, Tilly JL, Furuta I, Tsafriri A, Hsueh AJ (1994). Gonadotropin suppression of apoptosis in cultured preovulatory follicles: mediatory role of endogenous insulin-like growth factor I. Endocrinology.

[73] Sakurai M, Ohtake J, Ishikawa T, Tanemura K, Hoshino Y, Arima T, et al. Distribution and Y397 phosphorylation of focal adhesion kinase on follicular development in the mouse ovary. Cell Tissue Res. 2012;347(2):457–65. 10.1007/s00441-011-1307-2 22322421

[74] Boruah P, Shabbir S, Kulyar F (2021). Genome-wide transcriptome profiling uncovers differential miRNAs and lncRNAs in ovaries of Hu sheep at different developmental stages. Sci Rep.

[75] Cheng J, Zhao H, Chen N, Cao X, Chen H (2020). Population structure, genetic diversity, and selective signature of Chaka sheep revealed by whole genome sequencing. BMC Genomics.

[76] Zhang C, McKinsey T, Olson E. The transcriptional corepressor MITR is a signal-responsive inhibitor of myogenesis. Proc Natl Acad Sci U S A. 2001;98(13):7354–9. 10.1073/pnas.131198498 PMC34672 11390982

[77] Haberland M, Arnold MA, McAnally J, Phan D, Kim Y. Regulation of HDAC9 gene expression by MEF2 establishes a negative-feedback loop in the transcriptional circuitry of muscle differentiation. Mol Cell Biol. 2007;27(2):518–25. 10.1128/MCB.01415-06 PMC1800816 17101791

[78] Nakagawa O, Arnold M, Nakagawa M, Hamada H, Shelton J, Kusano H, et al. Centronuclear myopathy in mice lacking a novel muscle-specific protein kinase transcriptionally regulated by MEF2. Genes Dev. 2005;19(17):2066–77. 10.1101/gad.1338705 PMC1199576 16140986

