# Supplementary material for: Whole genome sequencing revealed genetic diversity, population structure, and selective signature of Panou Tibetan sheep
Source: BMC Genomics. 2023 Jan 28;24:50. doi: 10.1186/s12864-023-09146-2 (PMC9883975; doi:10.1186/s12864-023-09146-2)
Supplement: Supplementary file 4 — Additional file 4: Figure S1. Pairwise sequential Markovian coalescent (PSMC) analysis results for the Tibetan sheep inferred variations in Ne over the last 106 years. [file 12864_2023_9146_MOESM4_ESM.docx]

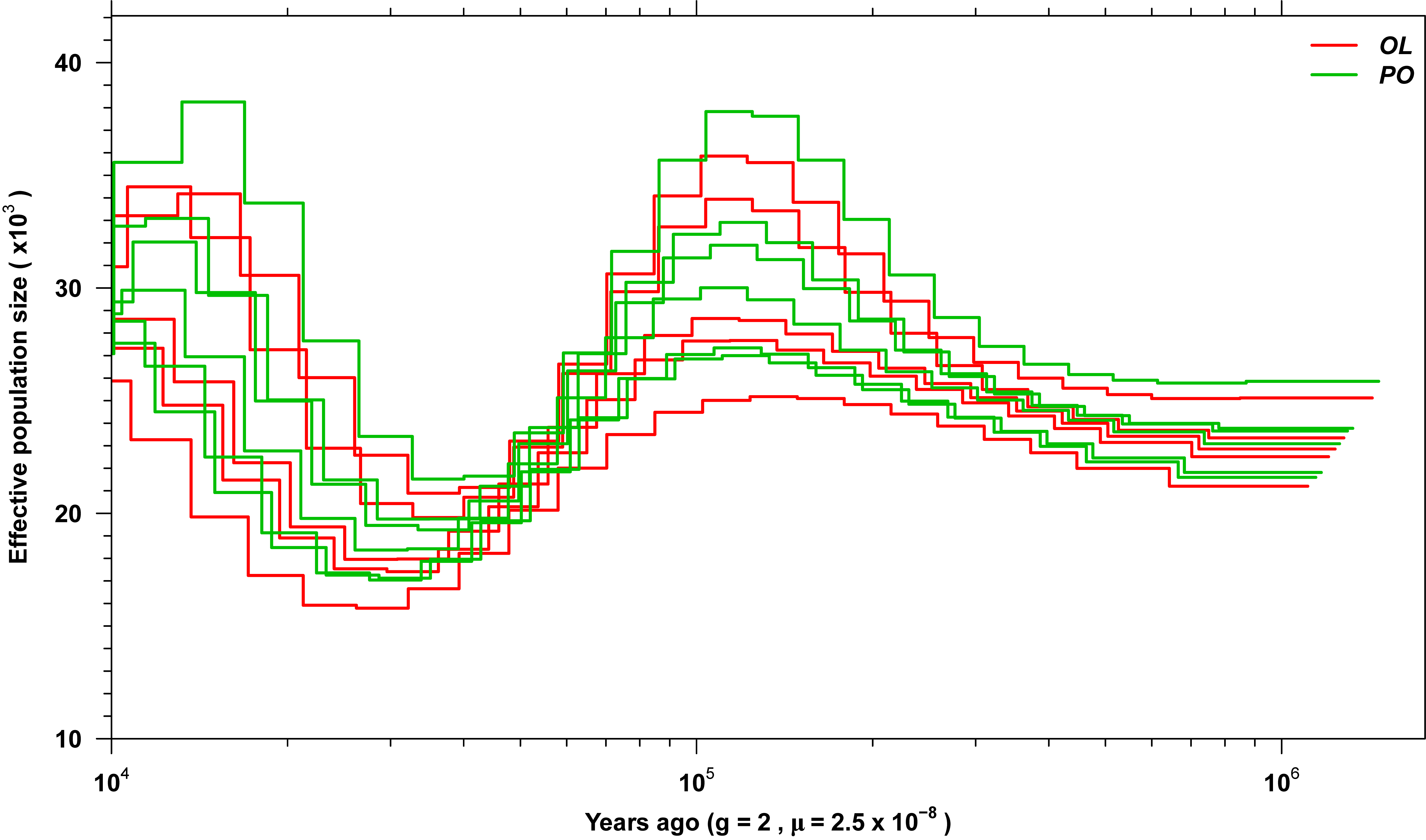


Figure S1. Pairwise sequential Markovian coalescent (PSMC) analysis results for the Tibetan sheep inferred variations in Ne over the last 10^6^ years.
