# Supplementary material for: Whole genome sequencing revealed genetic diversity, population structure, and selective signature of Panou Tibetan sheep
Source: BMC Genomics. 2023 Jan 28;24:50. doi: 10.1186/s12864-023-09146-2 (PMC9883975; doi:10.1186/s12864-023-09146-2)
Supplement: Supplementary file 7 — Additional file 7: Figure S2. Analysis of the signatures of positive selection in the genome of samples and genomic landscape of the Fst values. Figure S3. Analysis of the signatures of positive selection in the genome of samples and genomic landscape of the π ratio values. [file 12864_2023_9146_MOESM7_ESM.docx]

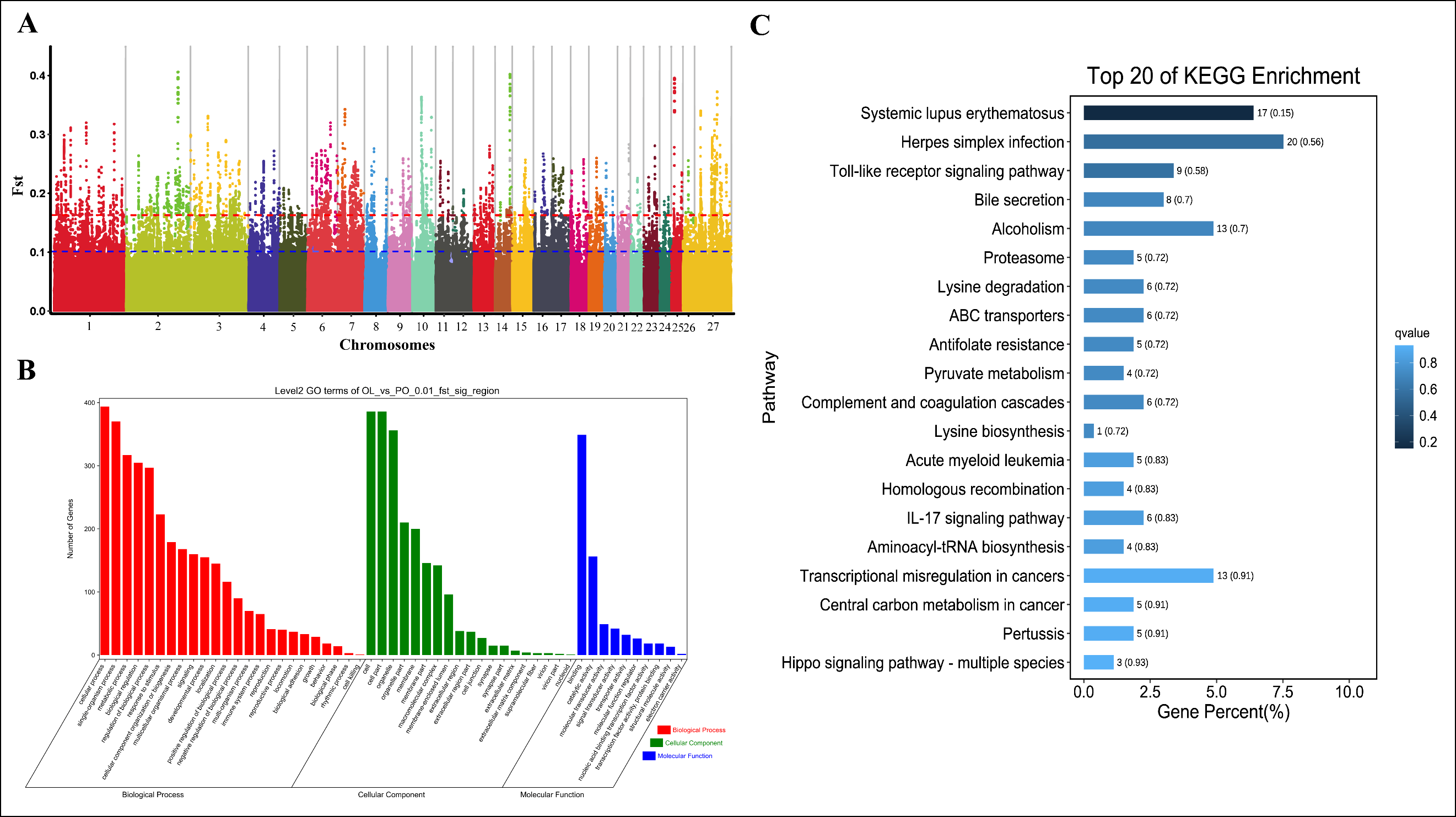


**Figure S2.** Analysis of the signatures of positive selection in the genome of samples and genomic landscape of the Fst values. **A.** The Fst on two breeds. **B.** GO heatmap selected analyzed by Fst. **C.** KEGG heatmap selected analyzed by Fst.


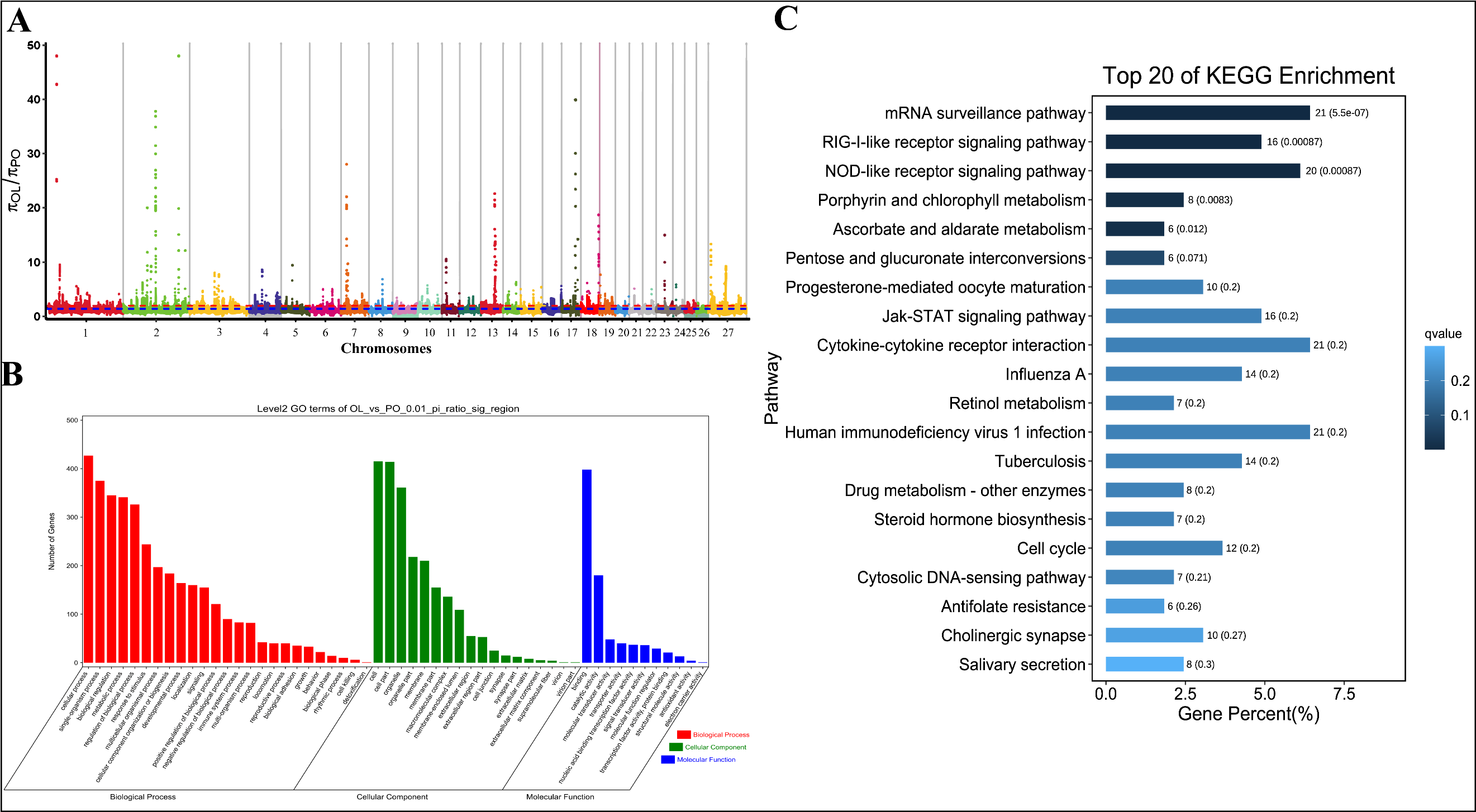


**Figure S3.** Analysis of the signatures of positive selection in the genome of samples and genomic landscape of the π ratio values. **A.** The π ratio on two breeds. **B.** GO heatmap selected analyzed by π ratio. **C.** KEGG heatmap selected analyzed by π ratio.
